# Exosome microRNAs in Amyotrophic Lateral Sclerosis: A Pilot Study

**DOI:** 10.3390/biom11081220

**Published:** 2021-08-16

**Authors:** Francesca Pregnolato, Lidia Cova, Alberto Doretti, Donatella Bardelli, Vincenzo Silani, Patrizia Bossolasco

**Affiliations:** 1 Experimental Laboratory of Immunological and Rheumatologic Researches, Istituto Auxologico Italiano, IRCCS, Cusano Milanino, 20095 Milan, Italy; francesca.pregnolato@gmail.com; 2 Department of Neurology-Stroke Unit and Laboratory of Neuroscience, Istituto Auxologico Italiano, IRCCS, 20149 Milan, Italy; l.cova@auxologico.it (L.C.); a.doretti@auxologico.it (A.D.); donatella_bardelli@yahoo.it (D.B.); vincenzo@silani.com (V.S.); 3 “Dino Ferrari” Center, Department of Pathophysiology and Transplantation, Università degli Studi di Milano, 20122 Milan, Italy; 4 “Aldo Ravelli” Center for Neurotechnology and Experimental Brain Therapeutics, Università degli Studi di Milano, 20122 Milan, Italy

**Keywords:** exosome, miRNA, amyotrophic lateral sclerosis, biomarkers, serum

## Abstract

The pathogenesis of amyotrophic lateral sclerosis (ALS), a lethal neurodegenerative disease, remains undisclosed. Mutations in ALS related genes have been identified, albeit the majority of cases are unmutated. Clinical pathology of ALS suggests a prion-like cell-to-cell diffusion of the disease possibly mediated by exosomes, small endocytic vesicles involved in the propagation of RNA molecules and proteins. In this pilot study, we focused on exosomal microRNAs (miRNAs), key regulators of many signaling pathways. We analyzed serum-derived exosomes from ALS patients in comparison with healthy donors. Exosomes were obtained by a commercial kit. Purification of miRNAs was performed using spin column chromatography and RNA was reverse transcribed into cDNA. All samples were run on the miRCURY LNA^TM^ Universal RT miRNA PCR Serum/Plasma Focus panel. An average of 29 miRNAs were detectable per sample. The supervised analysis did not identify any statistically significant difference among the groups indicating that none of the miRNA of our panel has a strong pathological role in ALS. However, selecting samples with the highest miRNA content, six biological processes shared across miRNAs through the intersection of the GO categories were identified. Our results, combined to those reported in the literature, indicated that further investigation is needed to elucidate the role of exosome-derived miRNA in ALS.

## 1. Introduction

MicroRNAs (miRNAs) are single-stranded non-coding RNAs involved in the regulation of gene expression and in the control of many cellular and metabolic pathways, such as cell migration, proliferation, and differentiation [1,2]. Previous gene expression analyses have enlightened that a large amount of miRNAs within the central nervous system (CNS) can cross the blood brain barrier and consequently be secreted in the cerebrospinal fluid and peripheral blood [3]. Detectable and quantifiable changes of these miRNA levels can act as a disease sentinel in patients and, therefore, may be suitable as disease-related biomarkers [4]. These small RNAs are abundant in body fluids and may be transported bounded to proteins or carried by vesicles [5,6]. Extracellular vesicles represent a peculiar way for cell-to-cell communication. Among them, exosomes with a diameter of 30–100 nm, are released from the cell by exocytosis through fusion of multivesicular bodies with the plasmatic membrane [7] circulating in the extracellular space adjacent to the site where they are discharged [8]. Consequently, they can release their content in the surrounding environment, or they can fuse with their target cells nearby. However, some of them move toward other anatomical districts through biological fluids and as a result they can be detected in several biological fluid, including serum, saliva, urine, and cerebrospinal fluid [9,10]. The internal composition of exosomes depends on their cellular origin. Indeed, these vesicles contain components specific of the host cell and behave as transmitters able to carry their content, particularly a substantial amount of miRNAs, from one cell to another [11]. Moreover, miRNAs encapsulated in circulating vesicles are stable in serum and, therefore, biologically active as gene expression modulators [12]. Different neural cell types release exosomes into the extracellular space, including neurons and astrocytes [13]. Exosome trafficking has been extensively characterized in tumor cells, but few data are available for the CNS, and especially in amyotrophic lateral sclerosis (ALS). In addition to their regulatory function, exosomes may be implicated in the local spread of neurodegenerative diseases when they cross the blood–brain barrier [14]. Indeed, in Alzheimer’s disease, these vesicles containing toxic proteins may lead to diffuse pathogenic amyloid deposition in the brain [15]. In Parkinson’s disease, alpha-synuclein has also been detected in extracellular vesicles whose level correlated with the severity of dementia. In ALS, mutant, as well as wild-type misfolded SOD1 protein, has been shown to be released in extracellular environment in association with vesicles [16]. Moreover, transcribed repeated RNA accumulation within RNA foci and its translation into toxic dipeptide repeat proteins (DPR) compromises repeat RNA metabolism and may, thus, exacerbate C9orf72-FTD/ALS pathologies in a vicious cycle [17]. To date, several studies reported distinct miRNA expression profiling in the serum of ALS patients compared to healthy donors, albeit few data are available for miRNA carried by circulating exosomes. ALS is an adult-onset disease characterized by the degeneration of motor neurons and leading to death within 3 to 5 years following disease onset [18]. In sporadic and several familiar ALS, the mislocalization of the transactive response DNA/RNA binding protein of 43 kDa (TDP-43) from the nucleus to the cytoplasm, as well as the formation of cytoplasmic aggregates of this protein represent a pathological hallmark [19]. However, no biomarkers reflecting the abnormal behavior of TDP-43 are available for early diagnosis and prognosis of the disease. Therefore, there is an increased need for useful markers in particular obtainable by a non-invasive manner such as by venipucture. To date, changes in the levels of the TDP-43, neurofilament light chain (NEFL), phosphorylated neurofilament heavy chain (pNEFH), and extracellular domain of the common neurotrophin receptor p75 (p75ECD) proteins are considered the best candidates as biomarkers of ALS [20]. Among miRNA biomarkers, an increase in miR-206 in the plasma of ALS patients has been shown to be a diagnostic and prognostic biomarker having a high correlation with Medical Research Council Score [21]. Two other possible prognostic markers, whose level is increased in serum plasma and cerebrospinal fluid of ALS patients are miR-9 [22,23,24], and miR-133b [25], as well as an increased association of miR-124-3p with spinal motor neuron-derived exosomes in SOD1G93A mice, even at the pre-symptomatic stage [26] albeit more studies are needed to confirm these data. Recently, two studies [27,28] reported differential expression between ALS patients and healthy controls of several miRNAs from plasma-derived neural-enriched extracellular vesicles, even though different miRNA were identified in the two papers.

In this pilot study, we aimed at providing evidence on feasibility of this study design and process. The primary outcome was to preliminary assess sensitive and reliable pathological biomarkers in the serum derived exosomes of ALS patients. To this aim, exosomes were isolated from ALS patient and healthy donor peripheral blood samples, and their miRNA content was evaluated using a real-time PCR instrument analysis. As pilot studies are not formally powered [29], our analysis focused on descriptive statistics and considerations on differential expression were not limited to statistically significant results but also extended to encouraging trends. Feasibility was explored with regard to process as recruitment rates, data collection, and experimental protocols (quality and efficiency of exosome isolation, and RNA extraction).

## 2. Materials and Methods

### 2.1. Participants and Samples

Peripheral blood samples (8 mL) were collected from 7 clinically diagnosed ALS patients (4 sporadic (S) and 3 mutated (M) for TDP-43) and 3 genetically unrelated healthy donors. Demographic and clinical features of the enrolled subjects are included in Table 1. Written informed consent was obtained from all participants and the study was approved by the ethical review board of IRCCS Istituto Auxologico Italiano in accordance with the Declaration of Helsinki. After collection, blood samples were allowed 30 min at room temperature for a clot to form and thereafter centrifuged at 4 °C for 15 min at 2000× *g*. Sera were stored at −80 °C until exosome isolation.

### 2.2. Exosome Isolation and Characterization

Exosomes were isolated using the miRCURY™ Exosome Isolation Kit-Serum and plasma (Exiqon, Copenhagen, Denmark). Briefly, samples were thawed on ice and centrifuged 5 min at 10,000× *g* to remove cell debris. Supernatant was transferred in a new vial and after addition of a precipitation buffer, samples were vortexed for some seconds to mix and incubated overnight at 4 °C. To precipitate exosomes, samples were centrifuged twice at 1500× *g* and pellets resuspended in a resuspension buffer for RNA extraction.

The quality of the isolation process was then verified by electron microscopy and western blot analysis. Freshly isolated exosomes were sent to the “Consorzio of Microscopy and image analysis (MIA)” to visualize the presence of exosomes by electron microscopy.

With regard to western blot analysis, the isolated exosomes were lysed using ice-cold Ripa buffer containing a cocktail of protease inhibitors (Sigma, St. Louis, MO, USA). Protein concentrations were determined using Pierce BCA protein assay (Thermofisher, Waltham, MA, USA). Ten ug of protein were loaded on a 4–12% sodium dodecyl sulfate polyacrylamide gel (Biorad, Berkeley, CA, USA). Proteins were then transferred to a nitrocellulose membrane. After blocking, the membrane was incubated with primary antibody anti-CD63 at 1:500 in 5% non-fat dry milk/PBS-Tween overnight at 4 °C. The secondary antibody was added at 1:10,000 (anti-mouse) Immunoreactive proteins were detected using ClarityTM Western ECL Substrate (Biorad). 

### 2.3. RNA Extraction

RNA was isolated using the miRCURY™ RNA Isolation Kit-Biofluids (Qiagen, Hilden, Germany) following manufacturer’s instructions. An RNA spike-in mix, including UniSp2 and UniSp4 (Qiagen), was added to each sample before isolation to ultimately detect differences in extraction efficiency. Moreover, 1 uL of Glycogen (Thermofisher), was added as carrier-RNA. The first step of this protocol consists of the lysis of exosomes membranes followed by precipitation of the proteins. RNA was then bounded to the resin of a spin-column by adding isopropanol to the transferred supernatant. After removing any remaining impurities using wash solutions, RNA was eluted with RNase free water. 

### 2.4. Real-Time PCR Panel Analysis of miRNAs

The samples were submitted to Qiagen Genome service, where miRNAs were profiled by miRCURY LNATM Universal RT miRNA PCR Serum/Plasma Focus panel (Exiqon, Cod EXESERAS031997).

All miRNAs were polyadenylated and reverse transcribed into cDNA in a single reaction step. 

The quality of the input RNA was checked by assessing the signal of two types of RNA spike-ins, the RNA included during the isolation phase (UniSp2, UniSp4) (Exiqon), and UniSp6 (Exiqon) that was added in the reverse transcription reaction. Finally, a DNA spike-in UniSp3 (Exiqon) was present in all panels in order to assess a potential inhibition at the qPCR level. As a negative control, a “no template” sample was included in the RT step.

At the end of the qPCR reaction, raw Cp values and melting points detected by the Lightcycler software (Roche, Basilea, Switzerland), were exported for the further analyses. Reactions with several melting points, or with melting points that are not within assay specifications, were removed from the dataset. Reactions with amplification efficiency below 1.6 were also removed. Finally, reactions giving Cp values that are within 5 Cp values of the negative controls reaction, were removed from the dataset. 

### 2.5. Data Analysis and Statistics

Raw Cq values were normalized by geometric averaging of multiple internal controls that were previously identified according to Normfinder algorithm [30]. A principal component analysis (PCA) was performed on the normalized dCq values in order to reduce the dimension of the dataset and thereby to provide insights on clustering and classification of samples. Relative quantification (RQ) for qPCR were calculated according to the method of 2^−^^ΔΔ^^Cq^. For miRNA detected in at least three sample per group, the differential expression analysis was evaluated using non-parametric Mann–Whitney test, and false discovery rate procedure was applied to adjust for multiplicity [31]. Four comparisons of differential expression were performed: ALS patients *versus* controls, mutated *versus* controls, sporadic *versus* controls, and mutated *versus* sporadic. 

Finally, potentially relevant miRNAs were further analyzed for their biological value using DIANA-miRPath v3.0 (http://www.microrna.gr/miRPathv3, accessed on 20 March 2021), an online free software suite developed to assess miRNA regulatory roles and to identify the controlled pathways [32].

The demographic and clinical characteristics of participants were summarized using descriptive statistics.

## 3. Results

### 3.1. Exosome Characterization

**Figure 1:**
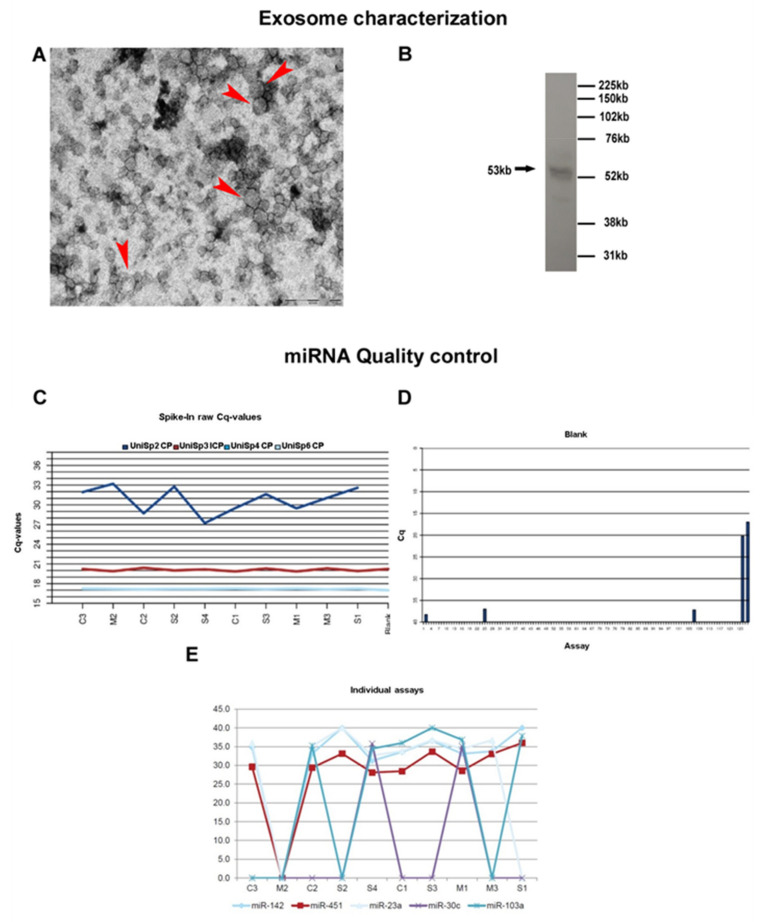
Exosome characterization and miRNA quality control. (**A**) Presence of exosomes in our samples was confirmed by electron microscopy (Arrow head) (scale bar 200nm) and (**B**) WB analysis. (**C**) The graph shows the sample quality control using spike-ins (RNA isolation control: UniSp2, UniSp4, UniSp5; cDNA synthesis control: UniSp6). On X axis the analyzed samples are reported (Controls: C1, C2, C3; Sporadic patients: S1, S2, S3, S4 and Mutated patients: M1, M2, M3) while Y axis shows the raw Cps obtained for the control assay. (**D**) Bar diagram showing raw Cp values for the negative control sample. Assays are reported on X axis while Cq levels are reported on Y axis. No signal was detected for the negative control. The positive controls on the plates (DNA and RNA spike ins) yields detectable signals as expected (far right). (**E**) The graph shows the average Cp levels for all the tested samples. On X axis the analyzed samples are reported while Y axis represent the Cp levels of miRNAs known to be expressed in a majority of sample types at a steady level.

Electron microscopy showing vesicles approximately of 100 nm (Figure 1A) and western blot analysis displaying a positivity for the CD63 antibody (Figure 1B) confirmed the presence of exosomes in the pellet of the processed samples.

Extraction efficiency was similar for all samples, and both reverse transcription and qPCR were successful (Figure 1C). The steady level, which is comparable to the blank purification, also shows that none of the samples contained inhibitors. Finally, no RNA contamination was detected (Figure 1D). To summarize, quality assessment monitoring for negative control and RNA spike-ins, indicated good technical performance of the profiling experiment. To evaluate the miRNA content of the samples, miR-103, miR-23a, and miR-30c, that are known to be expressed in a majority of sample types at a steady level were used. Expression of miR-451 and miR-142-3p was also evaluated, two miRNA highly expressed in blood cells and in thrombocytes. Samples that yield stronger signals than the average of the samples from this assay may contain a larger amount of blood or contamination with thrombocytes (Figure 1E). 

### 3.2. Overview of miRNA Profile

**Figure 2:**
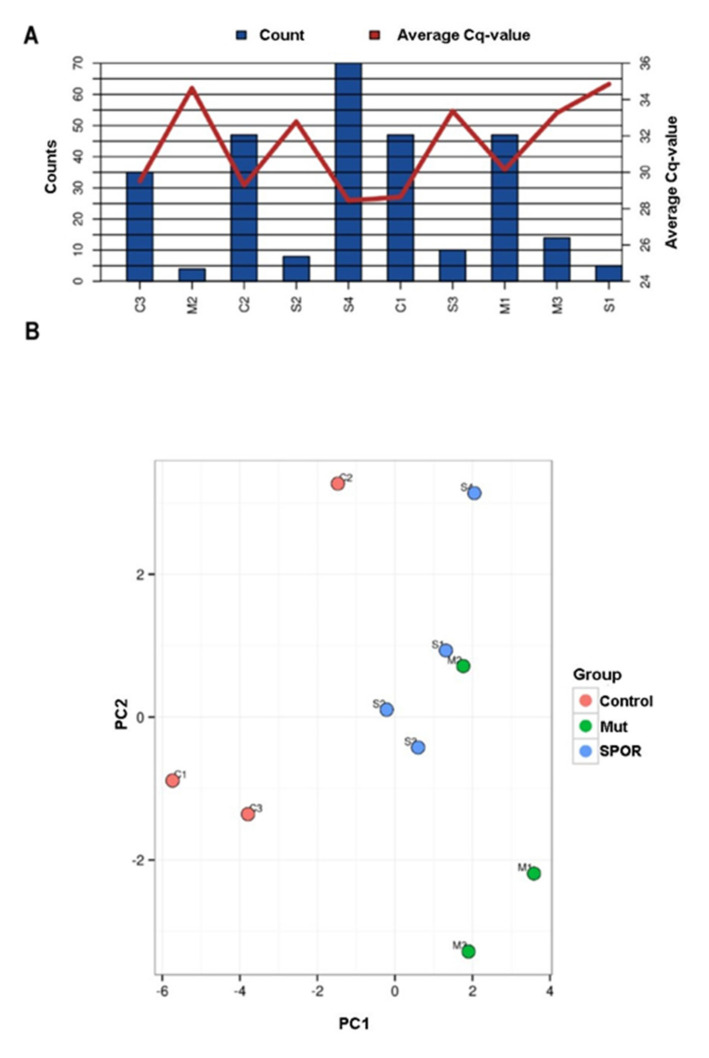
Number of detected miRNAs and PCA. (**A**) Graphical illustration of the miRNA content. The blue bars represent number of miRNAs detected in each sample and the red line shows the average Cq value for the commonly expressed miRNAs. On X axis the analyzed samples are reported (Controls: C1, C2, C3; Sporadic patients: S1, S2, S3, S4 and Mutated patients: M1, M2, M3). (**B**) PCA plot. The principal component analysis is performed on all samples, and on the top 25 miRNAs with highest standard deviation. The normalized (dCq) values have been used for the analysis. The largest component in the variation is plotted along the X axis and the second largest is plotted on the Y axis.

The overall miRNA content and their average Cq levels for all the samples are shown in Figure 2A. The miRNA content varied consistently, and five samples had very low call rates (M2, M3, S1, S2, S3). Indeed, among the 179 human miRNAs screened, only one was identified in all sample (miR-16-5p) and the mean number of assays detected per sample was 29. 

Nine miRNAs were identified for the normalization, six of which (has-miR-15a-5p, 451a, 21-5p, 16-5p, 223-3p, 142-3p) are known to be differentially regulated in ALS or in other neurodegenerative diseases even though in biofluids or biological compartments other than exosomes [33]. Geometric mean was consequentially calculated upon the remaining three miRNAs, has-miR-144-3p, 19b-3p, and 185-5p and used to normalize the raw Cq values. Then, a component analysis was performed on all samples and on the top 25 miRNAs with highest standard deviation: no clear clustering of groups or no outliers were detected (Figure 2B). Consistent with these findings, the differential expression analysis did not identify any statistically significant differences between groups. Therefore, we performed an analysis in a reduced sample set with the highest miRNA content and with patients and controls equally represented (S4, M1, M3, C1, C2, and C3). Due to the loss in statistical power, only ALS patients *versus* controls were compared and miRNA expression differences were only evaluated for trend and no statistical tests were performed.

The expression profile of the 10 miRNAs that were detectable in every six samples is indicated in Table 2.

### 3.3. Enrichment Analysis of Relevant miRNA

**Figure 3:**
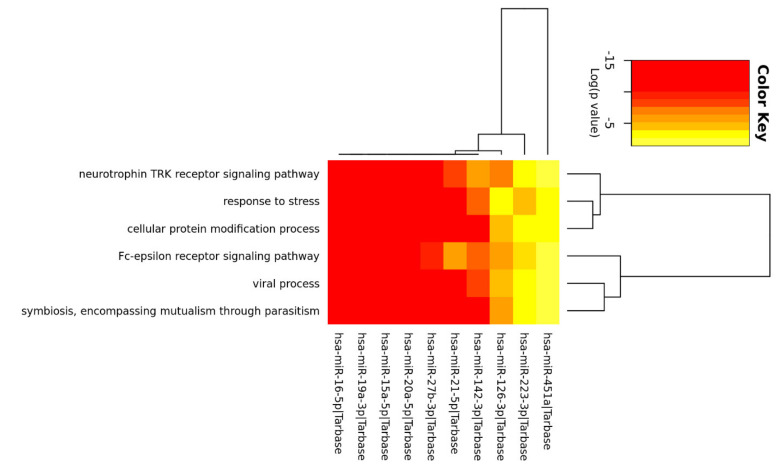
The heatmap depicts the level of enrichment in GO categories of the 10 miRNA that were all detectable in the subset of 6 samples when the intersection of the GO categories was considered.

A pathway analysis was then performed on these restricted group of 10 relevant miRNAs. Enrichment in GO categories enabled the identification of miRNA subclasses or GO terms that characterize similar miRNAs. The intersection of the GO categories identified 6 biological processes shared across miRNAs: neurotrophin TRK receptor signaling pathway, response to stress, cellular protein modification process, Fc-epsilon receptor signaling pathway, viral process, symbiosis, encompassing mutualism through parasitism (Figure 3).

## 4. Discussion

In this pilot study, we report an overview of the miRNA expression using a RT-qPCR analysis on serum-derived exosomes of ALS patients and healthy donor controls. We aimed to identify circulating markers for early diagnosis and therapeutic monitoring of ALS from routine blood sampling. The majority of the literature regarding the investigation of miRNA content in peripheral blood of ALS patients is carried out on whole body fluids (serum/plasma, cerebrospinal fluid, urine) [20,34,35,36]. On the contrary, we decided to investigate exosomal miRNA as these small vesicles possess a double-membrane structure resistant to the ribonuclease making their content more stable and refractory to degradation. In this pilot study, exosomes were obtained from peripheral blood samples of 7 patients and 3 healthy donors. As expected, due to the low sample size, no statistical differences were observed in the expression of any miRNA from ALS patients compared to healthy donors. Nevertheless, among the miRNA analyzed in our studies, some were previously reported to be differentially expressed in several biofluids of ALS patients (Table 3) albeit these studies were not performed on isolated exosomes. In two previous published studies [27,28], the different miRNA content of exosomes between ALS patients and healthy controls was similarly evaluated with minor procedural differences. Both laboratories used plasma as starting material and selected a neuron enriched fraction of extracellular vesicles selectively separated by mean of the L1 cell adhesion molecule antibody. Katsu et al. reported 13 up-regulated and 17 downregulated miRNAs in ALS compared to controls, while Banack et al. found a total of 8 differentially expressed miRNAs of which 5 upregulated and 3 down regulated with no overlapping of miRNA sequences detected. However, no adjustment for multiple testing was carried out in both studies, consequently leading to an inflated rate of false positive conclusions. In summary, the contrast with our results and divergence between the two studies may be explained from both a biological and a computational point of view that involves the variability of clinical samples, the different experimental procedures of vesicles isolation, as well as the distinct chips used to quantify the miRNA expression profile and the different normalization procedures.

As the miRNA content was generally low for the samples, we decided to evaluate miRNA expression differences for trend. In 6 selected samples, we found 10 miRNAs, involved in several biological processes, of which 6 were shared across miRNAs. In particular, 3 retrieved pathways are directly involved in the pathogenesis of ALS. The first identified was the “neurotrophin TRK receptor signaling” pathway. Secreted neurotrophins are powerful regulators of developing and adult nervous system [41] and play a role on neuronal survival and viability [42]. In particular, opposite roles on motor neurons (MNs) have been proposed for BDNF/TrkB [43]: potentiation of BDNF has been shown to enhance MN survival in vitro [44] however other studies have reported negative effects of BDNF/TrkB on MN survival making them more vulnerable to insults and enhancing their death by glutamate excitotoxicity, via activation of TrkB [45,46]. A second important pathway in the pathogenesis of ALS was identified, namely the “response to stress”. In response to a variety of stresses, cells generate stress granules, sites of mRNA storage, in order to promote cell survival. Recent studies have shown that some mutant proteins, such as the TDP-43 in ALS may co-localize with stress granules and disturb the dynamics of their assembly [47]. It has been hypothesized that excitotoxicity results in augmented protein oxidation, loss of proteasome activity, and endoplasmic reticulum (ER) stress in neural cells, thus contributing to MN death in ALS [48]. ER stress may also be driven by several stimuli, such as ageing, mutations, and oxidative stress, thus, disturbing proteostasis and inducing the misfolding and aggregation of proteins. In order to resolve ER stress, cells activate adaptive response through unfolded protein response (UPR). However, prolonged ER stress can induce apoptosis and neurogenerative diseases by affecting neurons and synaptic function, [49]. The third pathway in which our miRNAs were involved was “cellular protein modification process”. The accumulation of particular misfolded and aggregated proteins (ALS related proteins: TDP-43, SOD1, FUS) play a crucial role in the pathogenesis of the so called “protein misfolding disorder” ALS. Consequently, several aspects of this disorder can be modulated by post-translational modifications (PTM). Indeed, PTM can affect the protein homeostasis (turnover, aggregation, degradation). For instance, PTMs can promote protein stabilization and accumulation inside the cell enhancing its toxicity. On the other hand, PTMs can suppress protein toxicity by reducing its stabilization or increasing clearance [50]. Interestingly, a fourth pathway, named the viral process, may also be linked to ALS. Indeed, a possible contribution of viral infection has been suspected to be responsible for the pathogenesis of ALS [51]. In addition, an exosome-depended mechanism has been suggested to be involved in the transmission of misfolded proteins [52] and eventually enteroviruses [53,54,55,56].

In conclusion, our investigation of miRNA content into circulating exosomes did not revealed significant differences between ALS patients and healthy donors. However, to test statistical hypotheses was not of main interest of our research. Indeed, from a descriptive point of view and in line with the preliminary nature of a pilot study, the miRNAs we highlighted in terms of highest content and RQ were involved in fundamental ALS-related pathways suggesting possible connection between these specific miRNAs and neurodegeneration. However, the few data available in the literature regarding exosome-derived miRNA, as well as the discrepancies between the results of the different studies suggest that further investigations and larger sample size are needed. 

## Figures and Tables

**Table 1:** Summary of clinicopathological characteristics of patient cohorts.

	Sporadic (n = 4)	TDP-43 Mutated (n = 3)	Healthy Donors (n = 3)
Age at inclusion, mean (SD)	53 (17.8)	51.7 (22.9)	52 (30)
Gender, % M	100	100	100
Duration of the disease (months), median [IQR]	16 [19.5]	14 [19.5]	NA
Riluzole	Yes	Yes	NA
Onset % bulbar/spinal	25/75	0/100	NA
Fast/Slow	N	N	NA

N = No fast progression neither slow progression.

**Table 2:** Expression profile of the 10 miRNAs detectable in every six samples selected in a second step based on their best quality.

miRname	SD ALS pts	SD Controls	Average dCq ALS pts	Average dCq Controls	ddCq	RQ
hsa-miR-15a-5p	1.67	0.88	0.64	0.24	0.40	0.76
hsa-miR-451a	1.27	0.41	3.27	3.96	−0.69	1.61
hsa-miR-21-5p	1.11	0.99	0.51	−0.23	0.74	0.60
hsa-miR-16-5p	0.73	0.25	2.91	3.84	−0.92	1.90
hsa-miR-223-3p	1.35	0.70	−0.01	−0.15	0.14	0.91
hsa-miR-142-3p	1.03	0.20	1.43	−0.61	2.03	0.25
hsa-miR-126-3p	0.78	3.30	−0.87	−3.12	2.26	0.21
hsa-miR-19a-3p	0.69	0.58	−1.18	−0.54	−0.65	1.57
hsa-miR-20a-5p	0.53	0.19	−1.76	−0.51	−1.26	2.39
hsa-miR-27b-3p	1.00	0.99	−2.88	−4.44	1.56	0.34

RQ, relative quantity calculated as 2^−^^ΔΔ^^Cq^; SD, standard deviation.

**Table 3:** miRNA found to be differentially expressed in biofluids of ALS patients among the miRNA analyzed in our study.

miRname	Reference	ALS Type	Biofluid	Variation Reported	qPCR Normalization	Validated Target Genes
miR-15a-5p	Liguori et al., 2018 [37]	Sporadic	whole blood	decrease	miR-484	*hmga1, ucp2*
miR-451a	Liguori et al., 2018; [37]	Sporadic	whole blood	decrease	miR-484	*cdkn2d*
	Taguchi [38] and Wang, 2018	Sporadic, familial and mutated carriers	Serum	increase	RMA + DBAG algorithm (Affymetrix)	
miR-21-5p	Benigni et al., 2016 [39]	Sporadic	CSF	decrease	spiked in cel-miR39-3p, miR608, miR328-3p	
miR-16-5p	Liguori et al., 2018 [37]	Sporadic	whole blood	decrease	miR-484	*arhgdia, hdgf, hmga1, zyx*
miR-223-3p	Liguori et al., 2018 [37]	Sporadic	whole blood	decrease	miR-484	*cdkn2d*
miR-142-3p	Matamala et al., 2018 [40]	Sporadic	Serum	increase	Spiked in cel-miR39-3p	
miR-27b-3p	Liguori et al., 2018 [37]	Sporadic	whole blood	decrease	miR-484	

CSF, cerebrospinal fluid.

## Data Availability

The data presented in this study are available on request from the corresponding author and raw data will be archived in an Institutional Data Repository.
